# Effect of Simultaneous Sintering of Bioglass to a Zirconia Core on Properties and Bond Strength

**DOI:** 10.3390/ma14237107

**Published:** 2021-11-23

**Authors:** Noha Abdel Mawla El-Wassefy, Mutlu Özcan, Shaimaa Ahmed Abo El-Farag

**Affiliations:** 1 Faculty of Dentistry, Mansoura University, Mansoura 35516, Egypt; shaimaafarag@mans.edu.eg; 2 Faculty of Dentistry, Horus University, Damietta 10160, Egypt; 3 Center of Oral Medicine, Division of Dental Biomaterials, Clinic for Reconstructive Dentistry, University of Zürich, 8032 Zürich, Switzerland; mutluozcan@hotmail.com

**Keywords:** adhesion, zirconia, bioactive bioglass, bond strength, dental materials, resin cement, surface morphology, surface characterization

## Abstract

This study aimed to assess bioglass sintering to a zirconia core on surface properties and bonding strength to resin cement. Zirconia specimens were divided into four groups: G I: sintered; G II: bioglass modified zirconia (a bioglass slurry was sintered with zirconia at 1550 °C); G III: sandblasted using 50 μm Al_2_O_3_ particles; and G IV: Z-prime plus application. Surface morphology and chemical analysis were studied using a scanning electron microscope and energy-dispersive spectroscopy. Surface roughness was evaluated using a profilometer. Surface hardness was measured using an indentation tester. For the microshear bond strength test, resin cement cylinders were bonded to a zirconia surface. Half of the specimens were tested after 24 h; the other half were thermocycled (5–55 °C) for 1000 cycles. A shearing load was applied at a crosshead speed of 0.5 mm/min on a universal testing machine. Data were analyzed with ANOVA using SPSS software at (*p* < 0.05). Results: tThe mean surface roughness of G II was significantly higher than G I and G III. The microhardness of G II was significantly lower than all groups. For bond strength, there was no significant difference between groups II, III, and IV after thermocycling. Conclusions: Bioactive glass can increase the bond strength of zirconia to resin cement, and is comparable to sandblasting and Z-prime bonding agents.

## 1. Introduction

The development of high-strength zirconia is one of the recent advances in ceramic dental materials. Zirconia materials deliver speculatively higher fracture resistance and durability, as compared with porcelain and other nonmetallic alternatives [1]. Zirconia is a crystalline oxide of zirconium with similar mechanical properties to metals and a color resembling that of natural teeth. It has three different inherent crystalline structures, depending on pressure and thermal conditions, namely the monoclinic, tetragonal, and cubic phases [2].

Despite the superior mechanical properties of zirconia, one main obstacle is its weak bonding to different synthetic and nonsynthetic substrates due to its inert state. Thus, it cannot provide adequate bond strength following conventional cementation techniques [3].

Researchers have evaluated the strength and durability of different bonding protocols. A commonly used technique to increase the zirconia bond strength to resin cement is sandblasting (air abrasion). Using aluminum oxide particles in air abrasion is a conventionally used method to eliminate the superficial contaminated layers and increase micromechanical retention between composite cement and restoration [4]. However, there is clinical evidence that the combination of air abrasion at intermediate pressure and phosphate monomers containing primers and/or luting resins provide long-term durable bonding to zirconia ceramic and glass-infiltrated alumina under the humid and stressful oral conditions [4]. Long-term clinical studies verified that successful zirconia ceramic treated with air abrasion showed excellent clinical longevity [5].

Other protocols used for improvement of bond strength are tribo-chemical silica coating, glass micro-pearl silica-coating, selective infiltration etching, hot etching, laser irradiation, and PO_4_-ester monomer usage [6].

The bond strength of silica-based materials such as porcelain can be increased effectively by silane. However, a silane coupling agent alone is not efficient for zirconia bonding (non-silica-based restorations); these restorations require surface treatment before bonding. Likewise, hydrofluoric acid (HF) etching and silanization techniques are not effective for zirconia bonding enhancement [7].

Bioactive glass is well defined as a surface-reactive inorganic material. Crystalline phases can be produced by some bioactive glasses during thermal processing that reduce the degradation rate. Bioactive glasses can be used for enamel remineralization and bone regeneration [8,9]. The first generation of bioglass, developed in 1971, was silicate-based, consisting of 45% SiO_2_, 24.5% Na_2_O, 24.4% CaO, and 6% P_2_O_5_. Its composition is still among the most examined bioactive glass, and has been investigated for about 50 years [10]. The bioglass material can be etched more easily, and the presence of silica in its composition renders the surface more prone to bonding with resin cements. The bioglass process of bonding begins by a rapid cation exchange of Na^+^ and/or Ca^2+^ with H^+^ from solution, creating silanol bonds (Si–OH) on the glass surface, The pH of the solution increases, and a silica-rich (cation-depleted) region forms near the glass surface. Phosphate is also lost from the glass if present in the composition. Then, the high local pH leads to an attack of the silica network by OH^−^, breaking the Si–O–Si bonds. Soluble silica is lost in the form of Si(OH)_4_ to the solution, leaving more Si–OH (silanols) at the glass–solution interface.

Later, the condensation of Si–OH groups near the glass surface and repolymerization of the silica-rich layer occur. After that, the migration of Ca^2+^ and PO4^3−^ groups to the surface through the silica-rich layer and from the solution forms a film rich in amorphous CaO–P_2_O_5_ on the silica-rich layer. The final stage is an incorporation of hydroxyls and carbonate from solution and crystallization of the CaO–P_2_O_5_ film to HCA [11,12].

Another effective method used for surface activation of zirconia is the application of a primer that has acceptable cost and simple application. Primers that contain the composition of 10-methacryloxydecyl dihydrogen phosphate (MDP) and phosphate monomers can enhance zirconia’s bonding ability [13]. Improvement of resin bonding to zirconia mechanically can be obtained by air abrasion, while chemical bonding is achieved by adhesive monomers [14].

Measurement of bond strength is used to assess the effectiveness of an adhesive system and to subsequently predict its clinical application. The most commonly used types of bond-strength tests are of tensile and shear bond strengths. Researchers have described that cohesive fractures more frequently occur with shear bond strength rather than adhesive fractures. In clinical situations, cohesive failures are hardly detected in bonded restorations [15]. The stress distribution in the microshear bond (μSBS) test is more concentrated at the interface, which decreases the probability of cohesive failure in a material that does not characterize the accurate interfacial bond strength, and the μSBS test can differentiate between the effects of surface treatment methods better than the traditional shear bond strength tests [15].

Considering the sophistication of most procedures used to enhance zirconia/resin cement bond strength, this study was conducted to evaluate the effect of sintering bioglass together with soft-machined zirconia on the zirconia’s bonding ability to self-adhesive resin cements. The null hypothesis was that bioglass modification would nonsignificantly affect the zirconia/resin cement bond strength.

## 2. Materials and Methods

### 2.1. Specimens’ Preparation

#### 2.1.1. Specimen Machining and Sintering Process

For this in vitro study, a zirconia core (Nacera, Dortmund, Germany) was used. The composition of all materials utilized in the study is listed in Table 1. The zirconia was cut into plates with dimensions of 10 mm X 7 mm in length and 2 mm in thickness) using a low-speed diamond saw (Pico 155, Pace Technologies, Tucson, AZ, US), No. PI-BI-0217-004, under a water-cooling system.

#### 2.1.2. Bioglass Synthesis and Application

The bioglass particles were synthesized using the alkoxide sol–gel technique; silicon and phosphorus alkoxides were used with calcium hydroxide and sodium hydroxide. Deionized water and ethanol were used as solvents, and the resultant gel was aged for one week at 70 °C at a pH of 2. It was heat-treated at a temperature of 800 °C. Particles were spherical had an average size of 44 µm, containing 45 wt % SiO_2,_ 24.5 wt % Na_2_O, 24.4 wt % CaO, and 6 wt % P_2_O_5_ (Nanostream, 6th of October, Egypt).

Bioglass powder (500 μg) was mixed with 0.2 mL of distilled water on a glass slab with a spatula to obtain a slurry mix and smeared onto the zirconia plates’ surface with a medium-sized bonding brush (TPC Advanced Technology, Lawson St City of Industry, CA, USA). The slurry penetrated between the partially sintered zirconia particles by capillary action, and thus no changes in the thickness were considered. The sintering process occurred at 1550 °C, according to a cycle recommended by the manufacturer, in a sintering furnace (Luoyang Luwei Furnace Co., Ltd., Luoyang, China); this was marked as the bioglass-modified zirconia group (BMZ) G II.

#### 2.1.3. Surface Treatment and Specimen Grouping

For this in vitro study, zirconia plates were machined using low-speed diamond saw technology (Pico 155, Pace Technologies, Tucson, AZ, USA). Specimens were allocated into four equal groups (*n* = 28) according to treatment methods as follows: G I: control (sintered only); G II: bioglass-modified zirconia; G III: sintered then sandblasted; and G IV: sintered, sandblasted, then the Z-Prime Plus application. All study groups were sintered first at 1550 °C, as recommended by the manufacturer, in a sintering furnace (Luoyang, China). Sandblasting was done using a sandblaster apparatus (JNBP-2, Jianian Futong Medical Equipment Co. Ltd., Tianjin, China) using 50 μm Al^2^O^3^ particles at 2 bar pressure for 10 s, then rinsing in an ultrasonic cleaner device (MCS, CD4820, Codyson, China) for 60 min with distilled water and drying with an air syringe. For group IV, 28 zirconia plates were sintered, sandblasted, then treated with Z-Prime Plus (Bisco, Schaumburg, IL, USA) primer according to the manufacturer’s recommendation by applying 2 coats of Z-Prime Plus to the zirconia surface, and then drying for 3–5 s with an air syringe.

### 2.2. Material Testing

#### 2.2.1. Surface Morphology and Chemical Analysis

The surface morphology of all groups was compared using a scanning electron microscope (SEM, JSM-6510LV, JEOL, Tokyo, Japan). For this purpose, a representative specimen from each group was cleaned in an ultrasonic bath with 96% ethanol for 2 min and then air dried. Afterward, specimens were affixed on metallic stubs, coated (SPI-MODULETM, SPI Supplies, West Chester, PA, USA) by gold sputtering to render the surface conductive, and examined by SEM to detect surface topography at different magnifications. Energy-dispersive spectroscopy (EDS) (JSM-6510LV, JEOL, Tokyo, Japan) was employed to investigate the elemental composition of all groups. The working distance and voltage used during the surface scanning was 15 mm and 20 V, respectively.

#### 2.2.2. Measurement of Surface Roughness

Seven specimens were randomly selected from each group to measure the average surface roughness (Ra) by a profilometer (SURFTEST SJ-201, Mitutoyo Corp., Kawasaki, Japan). The stylus moved back and forth across each specimen; five readings were recorded, and the average roughness value (Ra) was calculated. The cut-off length was 0.8 mm, at a 0.5 mm/s scanning speed. The resolution of the recorded data was 0.01 µm.

#### 2.2.3. Measurement of Surface Hardness

Seven specimens were randomly selected from each group to measure the surface hardness at room temperature by the micro-Vickers hardness tester (JINAN PRCISION TESTING EQUIPMENT CO., Model HV-1000 LTD, Jinan, China). An indentation was made on the surface under a load of 200 g for 15 s using a diamond micro-indenter in the shape of a pyramid with a 136° angle between its faces. The Vickers hardness number (VHN) was automatically calculated using the following equation: VHN = 1854.4P/d^2^, where P is the applied load (g) and d is the average length of the indentations’ diagonals (μm).

#### 2.2.4. Microshear Bond Strength Testing

A low-viscosity self-adhesive resin composite cement (Charm SuperCem, Dentkist, Korea) was filled into a cut tube of a Nelaton catheter (Ultramed, Ultra for Medical Products Co., Assiout, Egypt, Lot 13A01N) with an interior diameter of 1.98 mm and a height of approximately 1 mm. The tube was securely held on the specimen’s surface using a tweezer to prevent the resin from seeping away from the defined area at the base. Then, the automixed self-adhesive resin cement was filled and cured for 20s (Bre.lux Power Unit; Bredent GmbH & Co., Senden, Germany) according to the manufacturer’s recommendation with 1400 mW/cm^2^ irradiance, a 430–480 nm wavelength, and a 10 mm tip diameter. The irradiance of the curing unit was calibrated by an Apoza radiometer (Apoza Enterprise, Chung-Cheng Rd, New Taipei City, Taiwan), and the resin was thoroughly cured through a clear tube. Subsequently, the specimens were stored in 37 °C water for 24 h. Half of the specimens were tested after 24 h, and the other half were aged and thermocycled for 1000 cycles between 5 °C and 55 °C water baths. The dwell time at each temperature was 30 s, with a transfer time of 15 s (Theromocycler, Robota, Alexandria, Egypt). The thermocycling conditions were based on the calculation that 1000 cycles would simulate the situation during one year in the oral cavity. After thermocycling, specimens were tempered to room temperature.

Prior to testing, specimens were inspected under (×40) magnification of an optical microscope for any defects. Specimens with remarked surface gaps, bubbles, or other noticeable defects were excluded. The zirconia–resin cement bonds were consequently tested.

A bonded specimen with composite micro-cylinders was mounted in a holding device and secured to the stationary part of a materials testing machine (Model LRX-plus; Lloyd Instruments Ltd., Fareham, UK). A loop made of a nitinol orthodontic wire with a diameter of 0.014 in was used to wrap the cylinder very close to its bonded base and aligned parallel to the loading axis of the upper mobile part of the universal testing machine. A shearing load was applied at a 0.5 mm/min crosshead speed. The chosen slow crosshead aimed to produce a shearing force during micro-cylinder debonding along the zirconia–resin interface. The debonding load was recorded in Newtons.

#### 2.2.5. Microshear bond strength calculation

To calculate the microshear bond strength, the failure load in (N) was divided by bonding area (mm^2^): τ = P/πr^2^, where:

- τ = bond strength (MPa);
- P = load at failure (N);
- π = 3.14;
- r = radius of the microcylinder.

The zirconia specimens were carefully examined for failure modes (adhesive, cohesive, or mixed) under a stereomicroscope (BS-3060C, Beijing, China) at magnification of ×40. The failure modes were classified as cohesive failure, which referred to a complete fracture within the ceramic or within the composite resin; adhesive failure; which meant a fracture between the ceramic (or composite resin) and bonding agent; and mixed fracture; which indicated a fracture involving two materials.

### 2.3. Statistical Analysis

All data were obtained as mean ± standard deviation and studied by an ANOVA test, using version 20.0 of SPSS software for Windows. When significant differences were found between the groups, a Bonferroni post hoc test was applied. The level of significance was set at *p* < 0.05.

## 3. Results

### 3.1. Surface Morphology and Chemical Analysis Results

**Figure 1:**
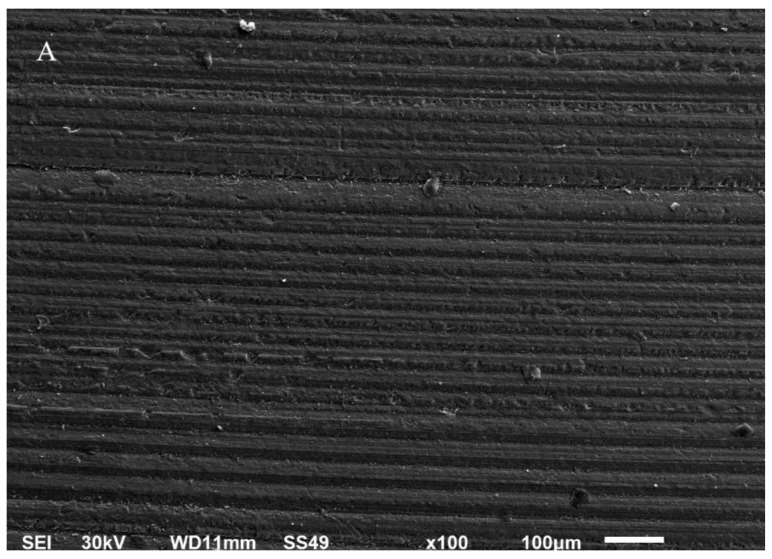

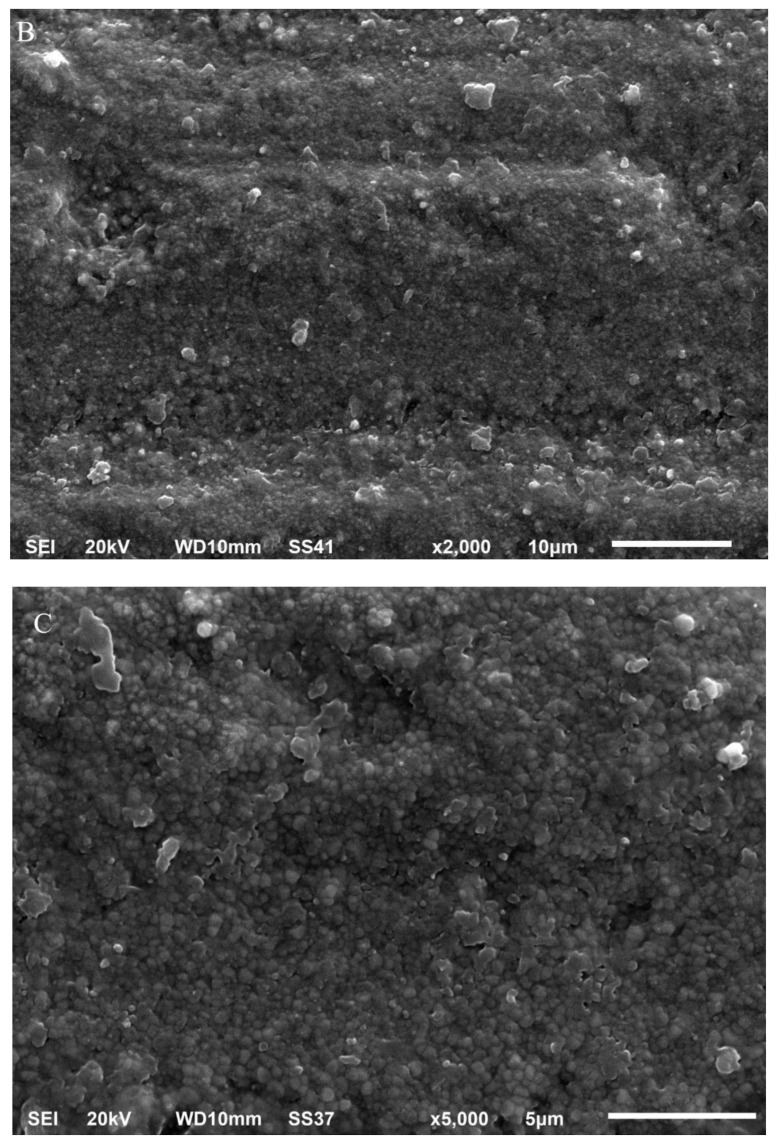

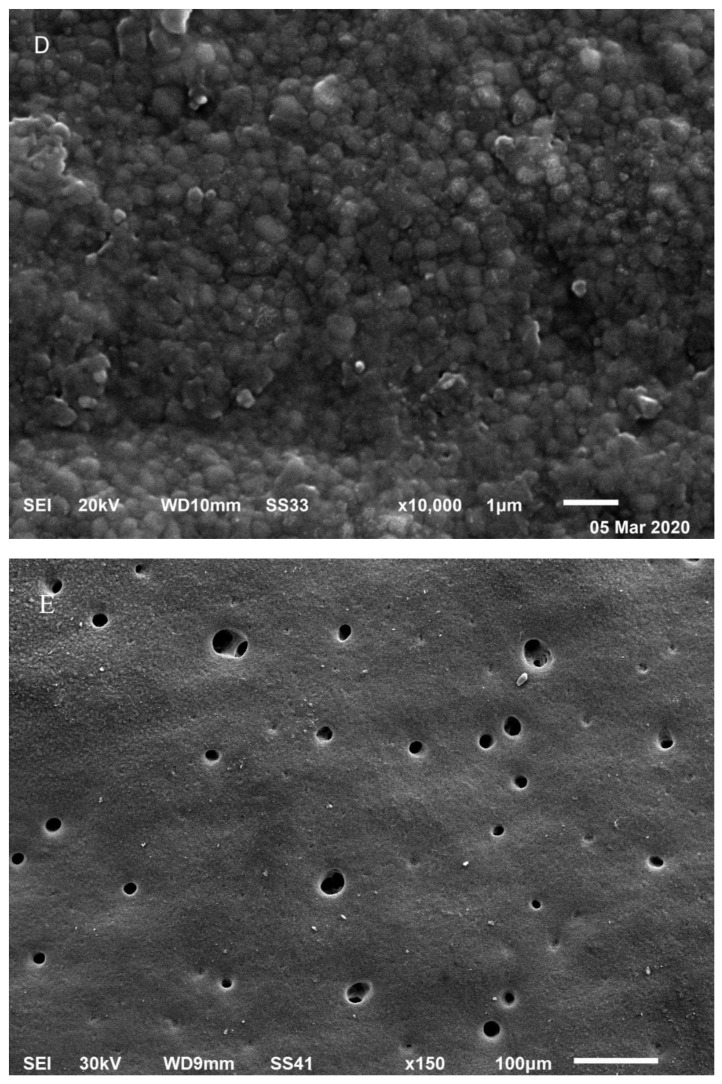

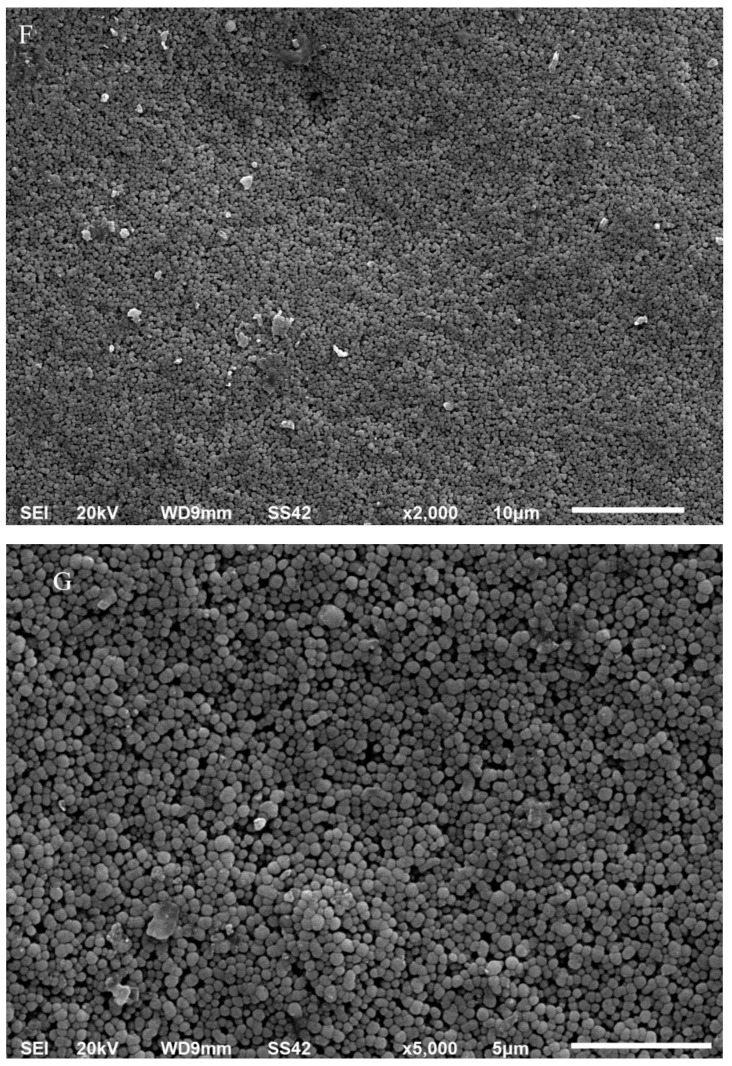

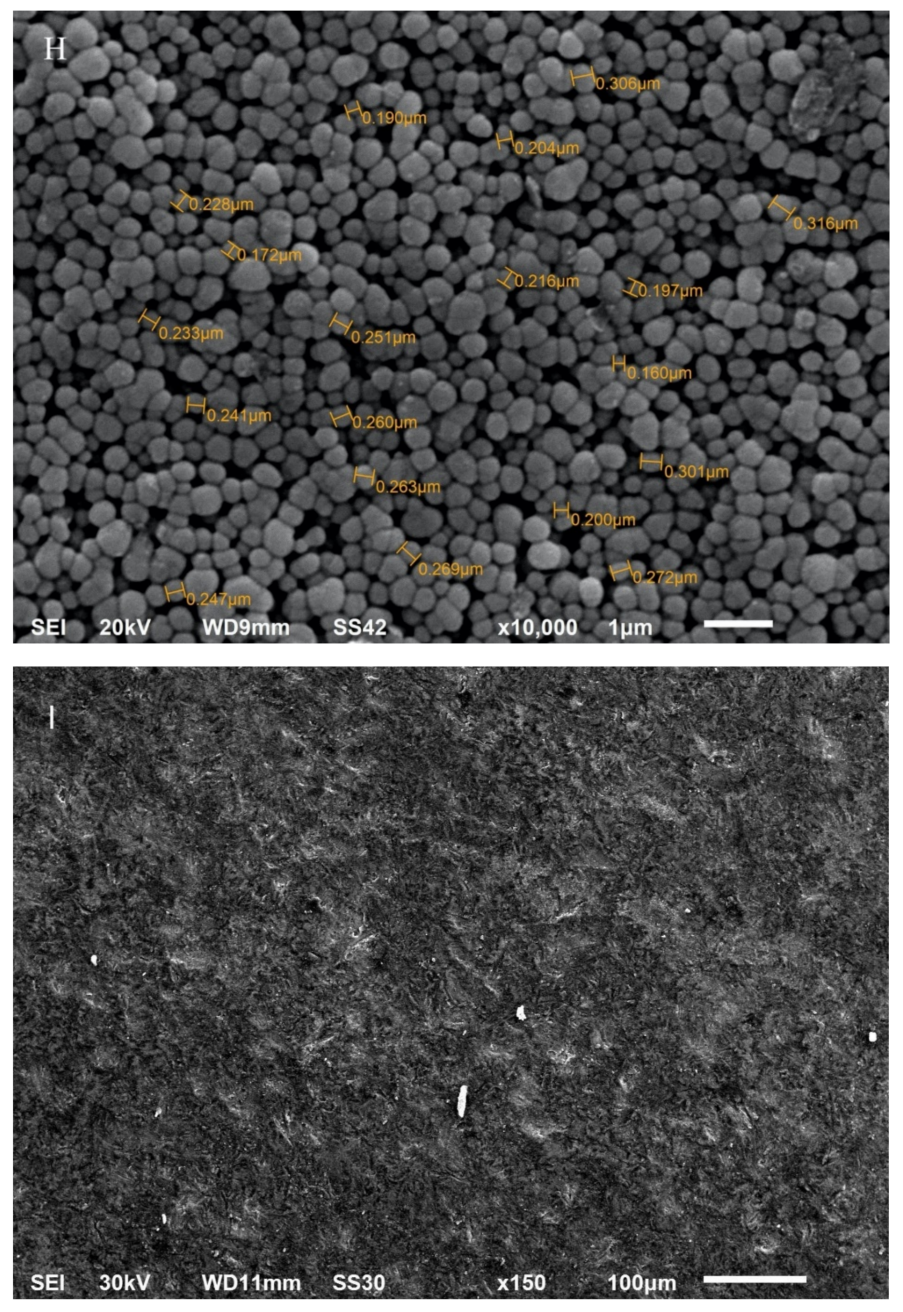

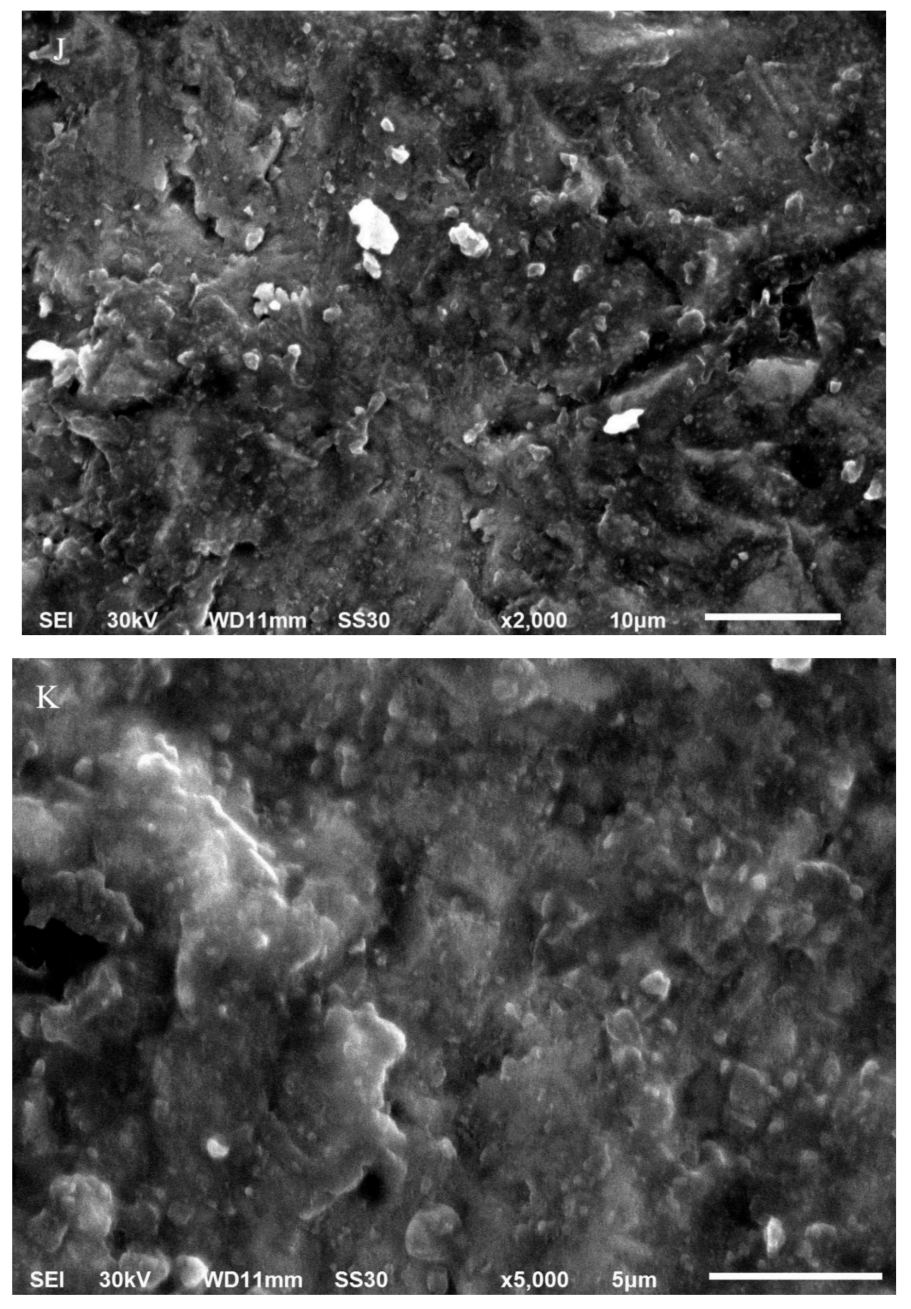

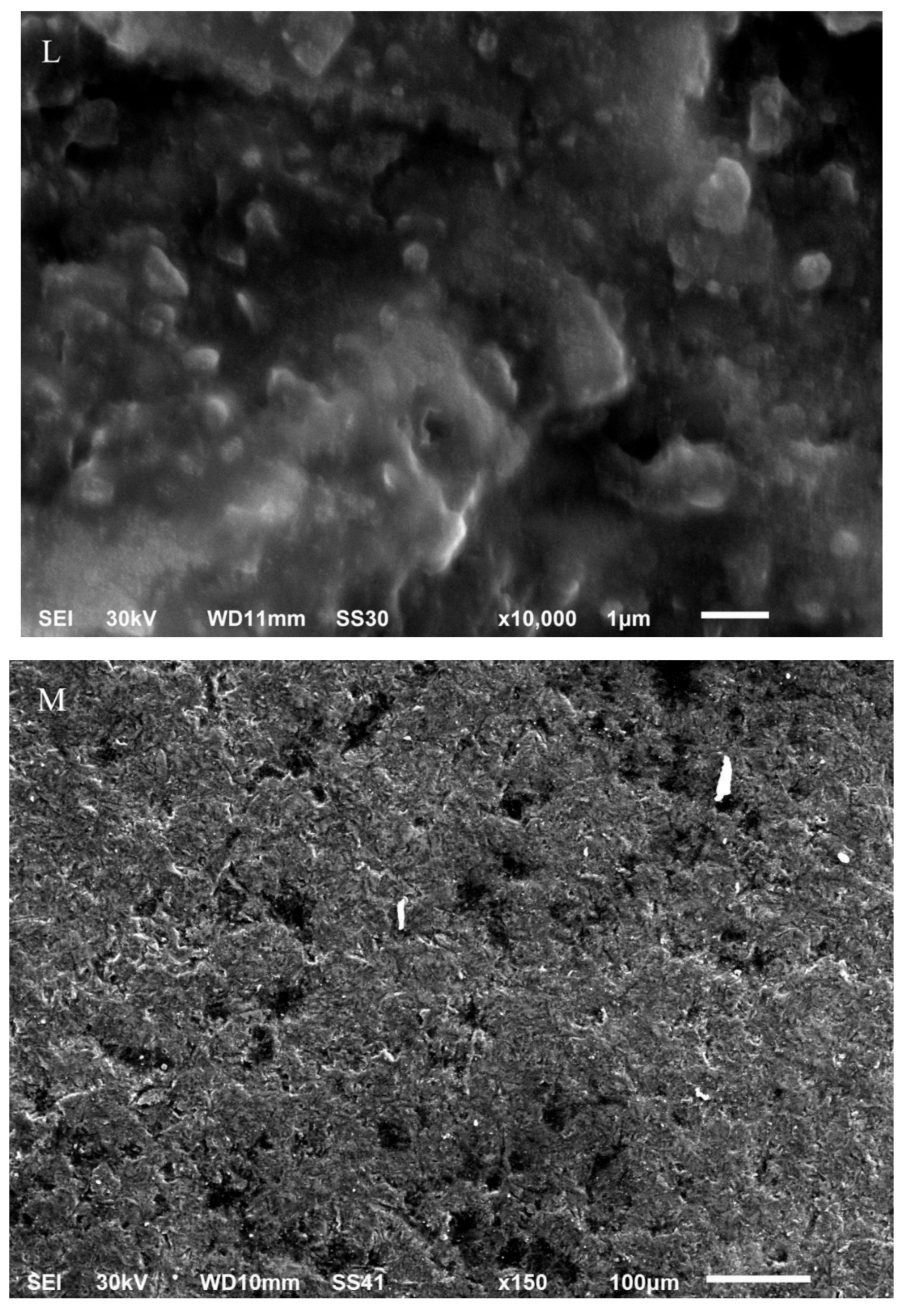

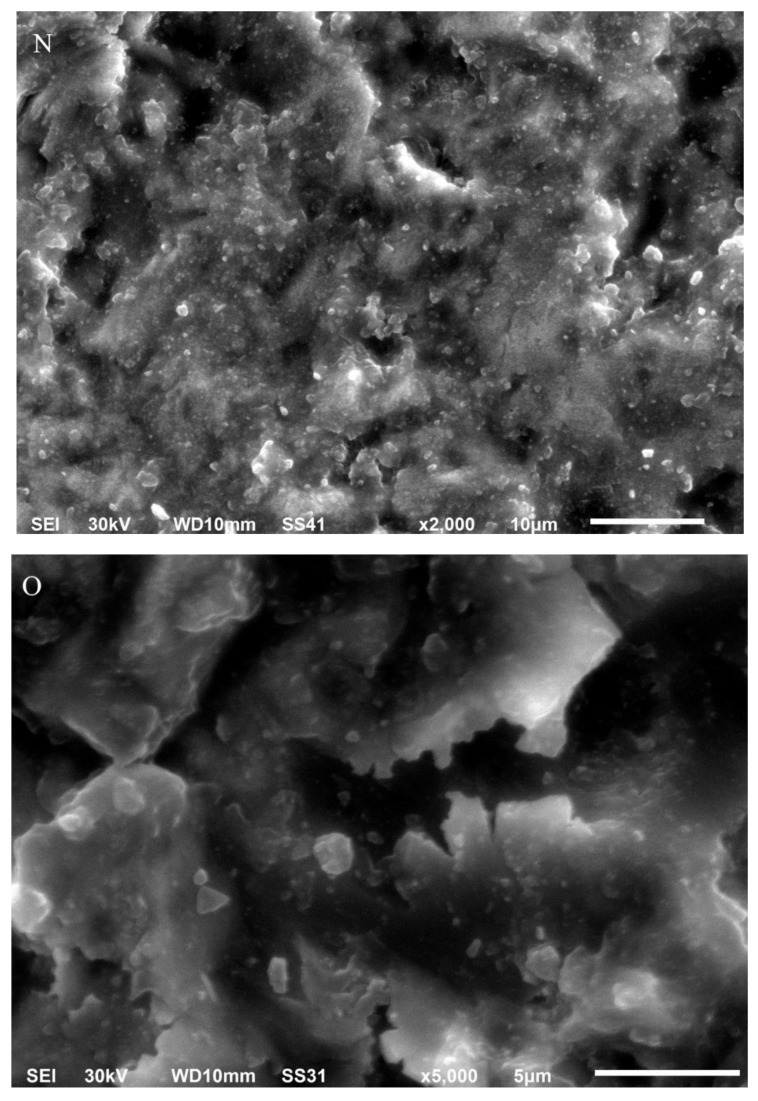

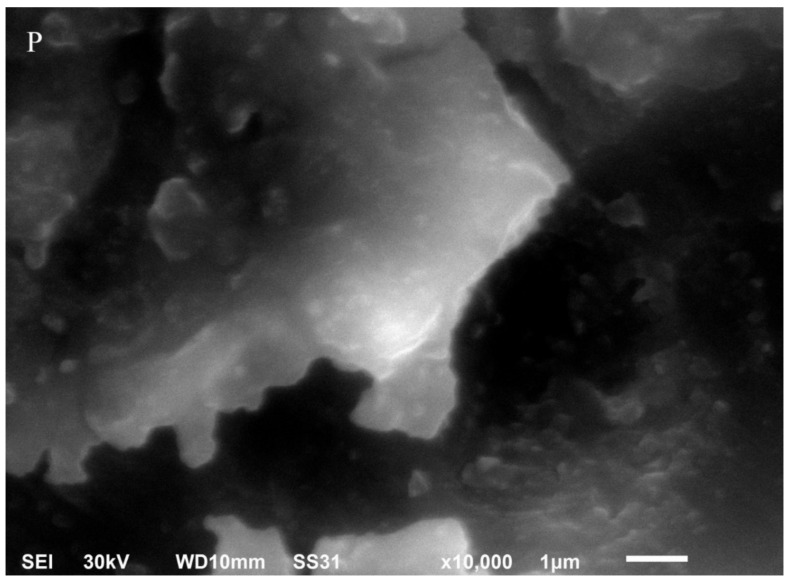
(**A**) Scanning electron photomicrograph of the sintered zirconia specimen with magnification ×100 showing the parallel grooves produced by the machine cutting disc. (**B**) Scanning electron photomicrograph of the sintered zirconia specimen with magnification ×2000 showing slight wavy depressions and elevations with condensed particles and negligible porosity. (**C**) Scanning electron photomicrograph of the sintered zirconia specimen with magnification ×5000 showing highly condensed, evenly sized granular particles with a slight wavy texture. (**D**) Scanning electron photomicrograph of the sintered zirconia specimen with magnification ×10,000 showing the sintered compacted granules. (**E**) Scanning electron photomicrograph of the bioglass-modified zirconia specimen with magnification ×150 showing different size porosities evenly distributed all over the specimen. (**F**) Scanning electron photomicrograph of the bioglass-modified zirconia specimen with magnification ×2000 showing homogeneous spherical compacted granules of nearly equal size. (**G**) Scanning electron photomicrograph of the bioglass-modified zirconia specimen with magnification ×5000 showing the bubbly granules with intervening spaces and scattered porosities. (**H**) Scanning electron photomicrograph of the bioglass-modified zirconia specimen with magnification ×10,000 showing measurements of spherical diameters. (**I**) Scanning electron photomicrograph of the sandblasted zirconia specimen with magnification ×150 showing evenly distributed surface texture all over the specimen. (**J**) Scanning electron photomicrograph of the sandblasted zirconia specimen with magnification ×2000 showing a surface texture with elevations and depressions, and fewer highly bright, irregular, loosely attached particles. (**K**) Scanning electron photomicrograph of the sandblasted zirconia specimen with magnification ×5000 showing a surface texture with elevations that appear irregular and larger than the specimen’s original granules. (**L**) Scanning electron photomicrograph of the sandblasted zirconia specimen with magnification ×10,000 showing a surface texture that appears irregular and larger than the specimen’s original granules. (**M**) Scanning electron photomicrograph of the Z-Prime zirconia specimen with magnification ×150 showing an evenly distributed finer texture all over the specimen with a considerable amount of evenly sized porosities. (**N**) Scanning electron photomicrograph of the Z-Prime zirconia specimen with magnification ×2000 showing an evenly distributed texture all over the specimen with a considerable number of porosities. (**O**) Scanning electron photomicrograph of the Z-Prime zirconia specimen with magnification ×5000 showing a rough irregular texture with some scattered spheroidal granules. (**P**) Scanning electron photomicrograph of the Z-Prime zirconia specimen with magnification ×10,000 showing rough, irregular, cleftlike elevations with some scattered spheroidal granules on top, and dark spacing underneath.

Scanning electron photomicrographs of all groups at different magnifications are shown in Figure 1A–P. At low magnification, the sintered zirconia surface shows the cutting marks of the low-speed disc, while the higher magnification shows the normal tetragonal granular structure of zirconia particles with very little intervening porosity, as the granules of sintered zirconia are stacked together with almost fused boundaries and negligible porosity (Figure 1A–D). The SE photomicrographs (Figure 1E–H) show that the bioglass sintered on the zirconia specimen had regularly distributed porosity on its surface. With higher magnification, the surface appears bubbly with spherical and spheroidal deposits with an average diameter of 0.224, and a considerable amount of porosity and intervening spaces are remarked between the spheres (Figure 1H). The SE photomicrographs (Figure 1I–L) show the surface morphology of the sandblasted group that appears altered than the sintered group, with adequately distributed surface texture and fewer porosities at higher magnifications. The SE photomicrographs (Figure 1M–P) show the surface morphology of the Z-Prime-treated group; the surface appears covered with homogeneously distributed fine texture materials, with some intervening porosities.

**Figure 2:**
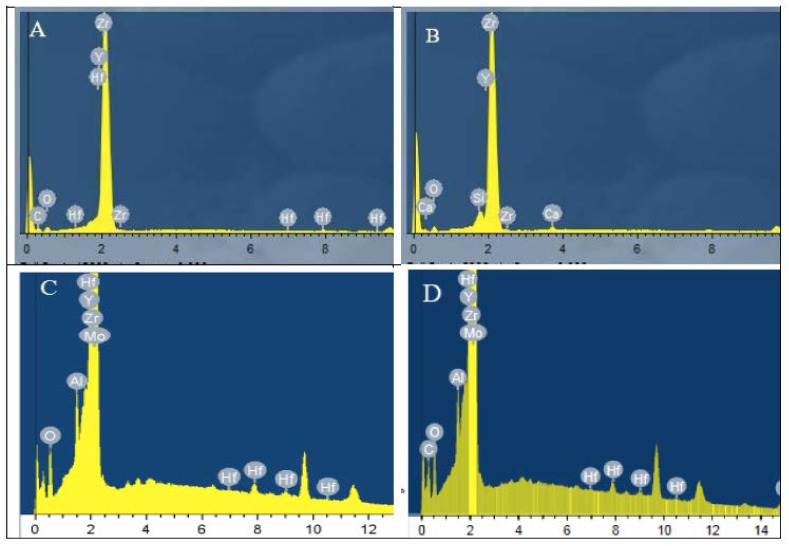
Representative energy-dispersive X-ray spectroscopy of the sintered zirconia group (**A**), the bioglass-modified zirconia group (**B**), the sandblasted zirconia group (**C**), and the Z-Prime zirconia group (**D**).

The energy-dispersive X-ray results for all groups are shown in Figure 2, and the elements are listed according to weight and atomic percentages in Table 2. The energy-dispersive X-ray analysis for the sintered zirconia (Figure 2A) showed the presence of Zr, C, O, Y, and Hf elements. The bioglass-modified zirconia group showed the presence of Zr, O, Si, Hf, and Ca elements (Figure 2B). The sandblasted group showed the presence of Zr, O, Y, Hf, and Al elements (Figure 2C). The Z-Prime group showed the presence of Zr, C, O, Y, Hf, and Al (Figure 2D).

### 3.2. Surface Roughness Results

It is shown in Table 3 that the lowest average surface roughness belonged to the sintered zirconia (0.45 μm ± 0.04), while the highest belonged to that of the bioglass-modified Zirconia group (3.21 μm ± 0.39). One-way ANOVA followed by a post hoc test showed that the bioglass and Z-Prime groups had statistically higher roughness values than the other groups (*p* = 0.00), while the sintered and sandblasted groups had an insignificant difference (*p* = 0.339).

### 3.3. Vickers Micro-Hardness Results

It is shown in Table 4 that the highest average Vickers microhardness (g·μm^−2^) belonged to the sandblasted zirconia group (1853.21 ± 201.44), and the lowest microhardness value belonged to the bioglass-modified zirconia (951.70 ± 170.81). One-way ANOVA followed by a post hoc test showed that the Z-Prime group was statistically nonsignificant compared to the sintered group (*p* = 0.622). The bioglass group showed a statistically significant lower value than the sintered and sandblasted groups (*p* = 0.000), and also showed a statistically significant lower value than the Z-Prime group at *p* = 0.032. The sandblasted group showed statistically significant higher values than the sintered and Z-Prime groups, where *p* = 0.020 and *p* = 0.013, respectively.

### 3.4. Microshear Bond Strength Results

Table 5 shows the means and standard deviations of the microshear bond strength (μSBS) in MPa for the different groups before and after thermocycling. Table 6 presents the results of the two-way ANOVA for the microshear bond strength, which showed a statistically significant difference between surface treatment and thermocycling at *p* = 0.000. Group I had a significantly lower microshear bond strength than all other study groups, before and after thermocycling. The μSBS did not significantly decrease after thermocycling in the sintered and sandblasted groups, while in the bioglass and Z-Prime groups, the μSBS significantly decreased after thermocycling, as shown in Table 5. The bioglass group showed a statistically higher microshear bond strength than the sintered group. There was no significant difference between the bioglass, sandblasted, and Z-Prime groups before thermocycling, with the Z-Prime group being higher in μSBS. In addition, there was no significant difference between the bioglass, sandblasted, and Z-Prime groups after thermocycling, with the bioglass group being higher in μSBS.

### 3.5. Mode of Failure Results

**Figure 3:**
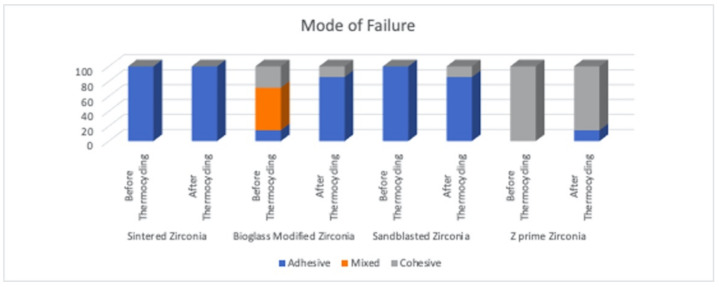
Column graph showing the different groups’ modes of failure after debonding and examination using a stereomicroscope.

Examining the debonded surfaces under a stereomicroscope showed that the mode of failure was prominently adhesive failure throughout all the examined specimens; however, the bioglass group before thermocycling showed mixed failure, and the Z-Prime group before thermocycling showed cohesive failure (Figure 3).

## 4. Discussion

In this in vitro study, bioglass material was sintered simultaneously with a zirconia substrate to form an adhesive layer, then characterized for its morphology, chemical structure, surface roughness, and hardness. Later, the microshear bond strength of bioglass-modified zirconia to resin cement was evaluated and compared to sandblasted and Z-Prime bonded groups, with the mechanical and chemical surface treatments being positive controls. In this study, the null hypothesis was rejected, as the bioglass-modified zirconia affected the resin cement/zirconia bond strength significantly.

In the present study, the creation of a continuous etchable bioglass layer on the zirconia surface was made by applying a bioglass slurry onto the soft-machined zirconia substrate; then the zirconia core and bioglass slurry were simultaneously sintered, creating a bioglass-modified zirconia structure. This eliminated the fear of debonding and delamination that could occur if the bioglass was applied secondary after zirconia sintering, as a separate layer of coating. In this study a self-etching, self-adhesive resin cement was used to produce an adhesive surface with the least required clinical steps, by omitting the etching step and decreasing the technique sensitivity.

This novel procedure of sintering bioglass and zirconia in the same furnace sintering cycle showed a homogeneous bubbly surface consisting of Zr, Si, and Ca, as confirmed by SEM and EDS. Scanning electron photomicrographs of the bioglass-modified group showed the development of a silica-rich glass layer with homogeneously distributed, evenly sized porosities (Figure 1E–H). This was in accordance with a study denoting that the sintering of a bioglass material formed silicate and calcium rich phases, as confirmed by EDS results [16]. It was also stated that the heating of bioglass slurry above its melting point during zirconia sintering would melt the glass, and the glassy matrix penetrated between the zirconia granules. In our study, a silica–calcium-rich layer with an average particle size of 0.224 μm was formed on the zirconia substrate as a glass surface layer. The bioglass surface layer was homogenous, even, and intact, with no cracks or defects [17].

In the current study, the bioglass-modified zirconia specimens showed a significantly higher surface roughness than the sandblasted group. As when the bioglass slurry was applied to the soft-machined zirconia surface and the sintering process took place, amounts of glass particles were melted and diffused between the zirconia granules, and therefore surface irregularities were seen on the surface. The presence of SiO_2_ and CaO sintered particles over the machined zirconia was confirmed by the EDS analysis, and is shown in Figure 2B and Table 2. It is clearly shown in the SE microphotograph of the bioglass group that it had a homogenous bubbly surface.

The hardness test presented very valuable evidence regarding the structural performance of the materials’ surface. The results of hardness testing showed that the bioglass-modified group had a significantly lower hardness value than the other study groups; this might be beneficial, as the silica layer was more prone to etching, and thus could provide better bonding to resin cements. Bioglass material was used in this study to form an etch-prone layer on the zirconia substrate, and then the bonding ability of bioglass/zirconia to resin cement was appraised as well. The Z-Prime group had an insignificantly lower hardness value than the sintered group, which might be attributed to the chemistry of the carboxylic monomers that contained conventional MDP. The sandblasted group had a significantly higher hardness value than all other groups; this might be attributed to the precipitation of Al_2_O_3_ particles within the abraded surface, as confirmed by the EDS chemical analysis results (Table 2), or could be due to the stress-induced transformation from the tetragonal to the monoclinic crystal phase, accompanied by an increase of the strength property [17,18].

Laboratory methods of bond strength testing are suitable predictors of dental restorations’ longevity, and they are claimed to assess bonding efficacy of adhesive systems and substrates. Bond-strength testing procedures are classified into macro and micro tests according to the size of the bonded surface area. In the current study, a microshear bond strength test was used for bond strength measurement that was relatively rapid and easily implemented, and one substrate could be used to evaluate numerous specimens [19].

Multistep adhesive resin cements have some constraints in their usage, as these materials are expensive, time-consuming, and technique-sensitive, and require multiple, complicated bonding procedures. Therefore, self-etching, self-adhesive resin cement (Charm SuperCem, Dentkist, Korea) was selected to be used in this in vitro study [20].

The Z-Prime Plus treatment group showed a higher statistically significant bond strength than the sintered group before and after thermocycling; this might be related to the chemistry of the Z-Prime material; as it bonded chemically to the zirconia structure. In addition, its surface morphology showed characteristic homogenous features and porosity. The Z-Prime Plus group also had significantly higher bond strength values due to its chemical structure, which contained conventional MDP and carboxylic monomers, which could chemically react with the zirconia oxide layer at the interface, as documented by the presence of a higher percentage of carbon by EDS [21]. The interfacial forces might have improved the wettability and chemical bonding to zirconia ceramics. In addition, MDP has an amphiphilic construction; the vinyl group, as the hydrophobic end, copolymerized with the resin monomer and the phosphate group as the hydrophilic end, could interact with the hydroxyl groups on the zirconia surface, enhancing the chemical affinity [13]. It seems that the synergistic effect between acidic MDP and carboxylic monomer was the most likely reason for having the maximum bond strength values. The results of this current study were in accordance with Shin et al. [22], who reported that the combined use of Z-Prime and air abrasion improved the bonding ability of resin cements to zirconia ceramic.

In our study, the sandblasted group was considered the positive control group, since this process is considered the most efficient zirconia treatment, as it produces micro-mechanical interlocking of the resin cement by increasing the zirconia’s surface roughness [3,23]. In this current work, the bond strength was significantly increased following sandblasting as compared to the sintered group; this showed agreement with previously mentioned studies’ results. Conversely, in another study, sandblasting did not increase the zirconia’s resin cement bond significantly [23]. A study considered that sandblasting inadequately roughened the surface of zirconia and did not attain a consistent bond [24]. Additionally, sandblasting has the shortcoming of forming shallow cracks and defects that reduce both fracture toughness and strength of zirconia restorations. Furthermore, sandblasting may disturb long-standing zirconia due to flaws and a transformation from the tetragonal to monoclinic crystalline phase [25].

In this study, 1000 cycles were performed to simulate intra-oral environmental aging conditions. One limitation of this study was the short storage period that did not allow for water saturation in the luting resin and at the bonding interfaces; hence, the long-term hydrolytic durability of the bonding interface was not tested. Therefore, long-term water storage for 150 days and 37,500 thermocycles until water saturation of bonded specimens should be performed in order to differentiate between clinically durable and nondurable ceramic bonding systems [26].

In the current study, there was a significantly higher microshear bond strength in the sandblasted, bioglass, and Z-Prime groups before thermocycling, which may be credited to the characteristics of surface roughness created in the bioglass and Z-Prime groups, as well as the silica contents documented in the chemical analysis of the bioglass layer. Z-Prime bonding agents containing MDP improved the bonding of resin cement to the zirconia ceramic [4]. After thermocycling, the bond strength was significantly decreased in groups II and IV. This might be attributed to the resin cement’s degradation and the water hydrolytic effect at the resin cement/zirconia interface. Moreover, the coefficient of thermal expansion mismatch between the bonded specimens and resin cement could result in stress during thermocycling [27].

Evaluating the failure mode of the resin–zirconia interface showed that most splits were cohesive in the Z-Prime group. The bioglass-modified zirconia group showed predominantly mixed and cohesive modes, while the frequency of adhesive failures increased in all other groups. There were neither cracks nor fractures at the bioglass surface, showing that the bond between the bioglass and zirconia was stronger than the bond between resin and bioglass.

## 5. Conclusions

From this in vitro study, it was possible to conclude that the use of bioactive bioglass to modify the zirconia surface during sintering was a feasible technique that could form a silica-rich layer. This sintered bioglass increased the zirconia’s surface roughness and reduced its microhardness. The use of bioactive bioglass to modify the zirconia surface could effectively increase the zirconia/resin cement bond strength, and could be an alternative to the already-established techniques, with the mode of failure being mixed before thermocycling.

Additional research is still compulsory, especially to assess the fracture resistance of zirconia crowns after sintering with bioglass materials in the intaglio surface. An in vivo study will be also conducted in the future.

## Figures and Tables

**Table 1:** The materials utilized in this study.

Materials	Batch	Composition	Manufacturer
Zirconia ceramic	5,054,089	Zirconium dioxide ZrO_2_ 3Y-TZP-A or 3Y-TZP	Nacera, Doceram Medical Ceramic Gmbh-Hesslingsweg 65–67, D-44309 Dortmund, Germany
Z-Prime Plus	1,800,001,455	BPDM, ethanol 75–85%, HEMA 5–10%, bis-GMA 5–10%, MDP 1–5%, proprietary (phosphate and carboxylate functional monomer)	Bisco Inc., Schaumburg, IL 60193, USA
Bioglass		24.5 wt % Na_2_O, 24.4 wt % CaO, 6 wt % P_2_O_5_, and 45 wt % SiO_2_	Nanostream, 6th of October, Egypt
SuperCem, Self-Etch Self-Adhesive Resin Cement	3,018,001	Base: silicon dioxide; barium glass, bis-GMA, triethyleneglycol dimethacrylate, diurethan-dimethacrylate; catalyst:silicon dioxide; barium glass, triethyleneglycol dimethacrylate, diurethan-dimethacrylate, champhorquione	DentKist, Inc, Eli-Dent Group S.P.A., Korea

**Table 2:** Energy-dispersive X-ray analysis results of atomic and weight percentages for the study groups.

Element	O	Si	Ca	Zr	Hf	Y	C	Al
Groups	At %	At%	At%	At%	At%	At%	At%	At%
Sintered	24.57			19	0.19	1.26	54.97	
BMZr	54.95	3.32	1.37	39.9	0.47			
Sandblasted	59.37			34.23	0.46	2.62		3.31
Z-prime	26.27			14.29	0.23	1.23	56.62	1.35

**Table 3:** Means and standard deviations of average surface roughness in μm of study groups.

Groups	Sintered_Zr	BM_Zr	Sandblasted_Zr	Z-Prime_Zr
Means + SD	0.45 ± 0.04 ^a^	3.21 ± 0.39 ^b^	0.61 ± 0.03 ^a^	2.08 ± 0.71 ^c^

Note: similar superscripted small letters denote a statistically nonsignificant difference at *p* = 0.05.

**Table 4:** Means and standard deviations of Vickers hardness (in g·μm^−2^) of the study groups.

Groups	Sintered_Zr	BM_Zr	Sandblasted_Zr	Z-Prime_Zr
Means + SD	1551.31 ± 115.55 ^a^	951.70 ± 170.81 ^b^	1853.21 ± 01.44) ^c^	1484.99 ± 319.31 ^a^

Note: similar superscripted small letters denote a statistically nonsignificant difference at *p* = 0.05.

**Table 5:** Means and standard deviations of microshear bond strength (MPa) for the different study groups before and after thermocycling.

Groups	GI Sintered_Zr	G II BM_Zr	G III Sandblasted_Zr	G IV Z-Prime_Zr
Before thermocycling	2.17 ± (0.92) ^a^	4.94 ± (0.63) ^b^	4.23 ± (0.84) ^bc^	5.08 ± (0.85) ^b^
After thermocycling	1.70 ± (0.97) ^a^	3.73 ± (1.03) ^c^	3.21 ± (0.92) ^c^	3.25 ± (0.45) ^c^

Note: similar superscripted small letters denote a statistically nonsignificant difference at *p* = 0.05.

**Table 6:** Two-way ANOVA of the microshear bond strength.

Source of Variation	Sum of Squares	Df	Mean Squares	F	*p*
Surface treatment	50.715	3	16.905	20.680	0.000
Thermocycling	17.967	1	17.967	21.979	0.000
Surface treatment * Thermocycling	3.368	3	1.123	1.373	0.262
Errors	39.238	48	0.817		
Total	812.632	56			

* For Interaction.
